# Keyword Index for Volume 106

**DOI:** 10.1038/bjc.2012.248

**Published:** 2012-06-05

20S proteasome 904

22Rv1 166

4EGI-1 1660

8-oxodG 344

ABCB1 1395

ABCG2 1320

abdominal cancer 490

*ABL1* 157

acidity 1280

acute myeloid leukaemia 475

adenoma 517

adenosine triphosphate-based chemotherapy response assay 53

adhesion 1535

Adjuvant! Online 1760

adjuvant 32

adjuvant chemotherapy 1239, 1268

advanced 1068

advanced breast cancer 440

advanced gastric cancer 1039

afatinib 6

age 1068, 1353

AIDS 447

Akt 1196

alcohol 592, 1881

ALDH1 846

ALK 1100

Amphiregulin 1406

anaemia 909

anal carcinoma and survival 756

anchorage-independent growth 553

angiogenesis 314, 896, 1089, 1153, 1495, 2004

angiotensin II-converting enzyme inhibitor 290

angiotensin II receptor blocker 290

animal exposure 966

*ANPEP* 157

antiangiogenesis 678

antifungal 1626

anti-HER2 therapy 673

apoptosis 314, 693, 711, 763, 839, 858, 1288, 1499, 1551, 1953

arginine 1481

arginine deiminase 324, 1481

argininosuccinate synthetase 324

aromatase inhibitor 1062, 1618

asbestos 575, 1016

aspirin 1564

ataxia telangiectasia 262

*ATM* 262

atrasentan 284

attitudes 1907

attributable fractions 1346

atypical endometrioid hyperplasia 1297

aurora kinase A 748, 1798

automated counting method 383

autophagy 324, 693, 711

avoidable deaths 1846

awareness 1907

AZD2281 468

*β*-catenin 1395

barasertib 858

Bcl-2 gene family 839

beta2-Microglobulin 1239

beta-catenin 517

bevacizumab 453, 468, 799, 1718, 1926

BH3 mimetic 711

biglycan 1214

bile duct cancer 1011

bile reflux 1342

biliary tumour 1011

BIM 763

biomarker 18, 85, 133, 148, 189, 199, 414, 460, 733, 955, 1039, 1153, 1160, 1506, 1718, 1989, 1960

bladder cancer 222, 366, 496, 1177, 1187, 1891

bone marrow stroma 1689

bortezomib 333

BRAF 939

brain metastases 25, 440, 1850

brain tumour 1702

*BRCA1/2* mutation carriers 2016

*BRCA1* 775, 1234, 1697

BRCA1/2 780

*BRCA2* 775

breast/ovarian cancer risk 2016

breast cancer 6, 25, 189, 248, 262, 375, 389, 397, 525, 592, 673, 780, 826, 916, 923, 1045, 1053, 1062, 1117, 1166, 1234, 1361, 1367, 1605, 1611, 1618, 1675, 1790, 1850, 1907, 1910, 1917

breast cancer risk factors 996

breast cancer subtype 1107

breast-conserving surgery 1160

breast density 996

breast neoplasms 1560

breast tumour cells 693

bystander effect 889

CA-125 633

cachexia 1583

CA IX 916

calcifications 525

calcium 1335

cancer cell resistance 1648

cancer cell viability 116

cancer drug targets 254

Cancer Referral Guidelines 436

cancer registry 1910

cancer screening 1361

cancer stem cell 99, 1320, 1512, 1997

cancer targets 1123

cap-dependent translation 1660

CAPIRI 453

carboplatin 70, 633, 1728

carboplatin resistance 482

carcinoma 1495

carcinomatous meningitis 440

case–control study 447, 1331, 1346

caspases 1499

Cav-1 1187

CCP score 1095

CD133 1997

CD151 923

CD24 846

CD44 846

CD46 1543

CD59 1543

CD55 1543

cell cycle 693

cell cycle genes 1095

cell death 711

cell death mechanisms 1766

CellSearch 508

cellular immunotherapy 1123

cervical cancer 1520, 1526, 1753

cervical cancer screening 1571

cervical cytology 817

cervical intraepithelial neoplasia 45, 817, 975, 1753

cetuximab 274, 793, 1406

CHAC1 189

checkpoint 429

chemical genomics 254

chemical proteomics 254

chemoembolisation 1274

chemokines 1306

chemoprevention 45

chemoradiotherapy 61

chemo-response 1967

chemosensitivity 333

chemotherapy 274, 651, 658, 896, 947, 988, 1033, 1076, 1591, 1605, 1934

chemotherapy resistance 1901

childhood cancer 982

childhood infections 1331

childhood leukaemia 1556

*Chlamydophila psittaci* 966

cholangiocarcinoma 1583

chrysotile 575

circulating-free DNA 375

circulating tumour cells 375, 508, 939, 1605, 1790

circulating tumour microemboli 508

cisplatin 460, 658, 1027

c-Jun 1997

CK-19mRNA 1917

class predictor 126

clinical practice 1760

clonal growth 1506

clusterin 1945

CML 1742, 1772

CNS metastasis 397

CNS symptoms 839

coeliac disease 217

cohort 210, 217, 596, 1004, 1011, 1842

colon cancer 748, 1239, 1268, 1395

colon cancer cell lines 141

Colorectal 1386, 1718, 1907

colorectal adenoma 608

colorectal cancer 18, 126, 133, 141, 227, 517, 793, 799, 805, 931, 1320, 1335, 1374, 1431, 1499, 1564, 1713, 1722, 1735, 1826, 1833, 1875, 1926, 2010

colorectal cancer liver metastases 1274

colorectal cancer screening 1424

combination chemotherapy 1728

combination therapy 107, 1386, 1742, 1779

common variants 389

comorbidity 1353, 1860

competing risks 1846

complex intervention 826

confidence interval 1846

conformal radiotherapy 61

controlled clinical trials 1259

copy number 748

cost 1045

cost-effectiveness 805, 1100, 1571

C-reactive protein 702

crizotinib 1100

crocidolite 1016

CRP 1866

CXCL12 1306, 1520

CXCR2 1833

CXCR4 1306, 1520

CXCR7 1520

cyclin-dependent kinase 133

cytochrome-*c* 889

cytokines 1134

cytology 975

cytotoxic 1379

D310 mutation 1506

dacarbazine 85

dasatinib 85

data integration 1107

DCE-MRI 1926

DCIS 1160, 1506, 1611

decision 1045

decision aid 1053

decision-making 1053

definitions 1262

definitive RCT 826

delayed infection 1556

deletion 719

dendritic cells 546

depsipeptide 116

detection 436, 1605

diabetes 1374

diagnosis 733, 904, 909, 982, 1068, 1431, 1556, 1940

diagnostic intervals 1262

diet 603, 608, 1891

dietary fat 596

diffusion-weighted magnetic resonance imaging 1638

diindolylmethane (DIM) 45

disease-free survival 955

disease progression 1249

disseminated tumour cells 375

DMXAA 1134

DNA damage response 429

DNA methylation 248, 414, 585

DNase 206

docetaxel 858, 1469

dormancy 375

dose escalation 678, 1598

double-strand DNA repair 1460

doublet 658

doxorubicin 916

driver-network 1107

drug discovery 254

drug–drug interaction 1772

drug resistance 1089, 1901

drug sensitivity 116, 867

DSC1 756

dual erbB protein kinase inhibitor family 666

ductal carcinoma *in situ* 1611, 1675

duodeno-gastro-oesophageal reflux 1342

dynamic equilibrium 1512

E7080 1598

early breast cancer 1760

early detection of cancer 975

early diagnosis 1262

EBNA1 206

EBV 1980

E-cadherin 756

education of patients 651

EEF1A1 166

EEF1A2 166

effectiveness 1045

effectiveness of the QoL system 826

EGFR HER2 719

EGFR PharmDx 883

EGFRvIII 883

eIF4F complex 1660

elderly 274, 1353

electromagnetic fields 307

ELISA 1495

EML4–ALK 763

EMT 1196, 1735

endometrial cancer 1551, 1682

endometrioid endometrial cancer 1297

endothelin axis 284

enzautaurin 867

EPIC 1866

epidemiologic methods 569

epidemiologic studies 970

epidemiology 1860

epidermal growth factor receptor 666, 883, 1100, 1196, 1367, 1406, 1520

epigenetically silenced 482

epigenetics 248, 414, 1446

epinephrine 460

Epiregulin 1406

epithelial cells 1205

epithelial mesenchymal transition 1512

epothilones 70

Epstein–Barr virus 206

ER*α* 1682

ER positive 1798

erythropoiesis-stimulating agents 1249

erythropoietin receptor 1249

ESFT cell lines 1288

ethnicity 1361

everolimus 876, 1039

evolutionary game theory 174

Ewing's sarcoma family of tumours 1123, 1224

exemestane 1062

exon array 538

expected survival 1854

expression profiling 538, 1095

extracellular 1453

extracellular signal-regulated protein kinase (ERK) 1997

eye 1171

EZH2 243

FADD 1989

faecal immunochemical test 805

faecal occult blood tests 1424

family history 780

Fas/Fas L upregulation 1288

FBXW7 182

FDG-PET/CT 1926

fertility 1053

FFPE 538

*FGFR2* 727

FGFR3 1406

fine-needle aspiration biopsy 562

first-in-human 666

five subtypes 923

flexible sigmoidoscopy 805

FLT3 475

FLT3-ITD 475

fluconazole 1626

FOBT 805

FOLFIRI 453

FOLFOX therapy 126

follow-up 1611

food supplement 45

FUS-DDIT3 1976

fusogenic glycoprotein 496

gallstones 1011

gamma-glutamyltransferase 1551

gamma-glutamyltranspeptidase 1551

gastric adenocarcinoma 733

gastric cancer 210, 585, 685, 727, 740, 1469, 1535, 1591, 1668

gastric resection 1342

gastric surgery 1342

G_D2_ 1123

gefitinib 1148, 1196

gemcitabine 1934

geminin 1798

gene amplification 727

gene-body 248

gene expression 867, 1648, 1967

gene mutations 1648

genetic testing 1234

gene transfer 1123

genomic stability 1297

genotype 1591

γH2AX 1807

Glasgow Prognostic Score 279

glioblastoma 1702

glioma 538

glucose 227

glutathione 1551

GPER 1682

GPRD 233

growth factors 1033, 2004

*γ*-secretase inhibitor 1953

GSK-3*β* 1196

HA14-1 711

haeme iron 608

HDAC-inhibitor 116, 1682

head and neck cancer 1526

head–neck cancer radiotherapy 846

health personnel 651

health services research 1021

Hedgehog signalling pathway 1177

heparanase 1486

hepatocellular carcinoma 307, 1439

hepatocellular carcinoma metastasis 1486

HER2 6, 1367, 1453

Her2neu 1543

HER2+ 25, 32

hereditability 389

hereditary 1460

histamine-2-receptor antagonists 233

histone deacetylase inhibitor 77, 107

histone methyltransferase 243

HIV 447

HLA class I 1980

HMW-MAA 939

homeostasis model assessment-insulin resistance 227

hormone receptor-positive 1760

HPV vaccination 1571

human 1224

human 8-oxoguanine DNA glycosylase 344

human epidermal growth factor receptor 2 (HER-2)-positive breast cancer 440

human papillomavirus (HPV) 45, 222, 358, 817, 975, 1526, 1753

human polyomavirus 222

hybrid capture 975

hydroxyapatite 525

hypergastrinaemia 233

Hypertension 1718

hypoxia 314, 904, 916

hypoxia hypoxia-inducible factor-1 1638

ICR10 883

IFN-*α* 284

IgA 206

IGF1R 1367

IGFBP-3 1004

IGF-I 1004

IHC4 1760

*IL23R* 517

IL-6 1866

IL-8 1833

imaging biomarker 619

Imatinib 1772

immortalisation 1205

immune-modulation 896

immunodeficiency 447

immunohistochemistry 141, 883, 947, 1033, 1926

immunotherapy 793, 896, 988

imputation 1068

incidence 962, 970, 1335, 1753, 1850

India 962

indole-3-carbinol (I3C) 45, 333

indoleamine 2,3-dioxygenase 1 141

inequalities 1361

infections 1556, 1626

inflammation 199, 217, 1439, 1866, 2010

inflammatory markers 1424

iNOS 889

*in situ* hybridisation 1790

insulin resistance 1335

integrin-*β*1 846

integrin signalling 1535

inter-conversion 1512

interferon-*α* 1742

interleukin-6 1583

internet 651

intolerance 1475

intragenic 248

intraperitoneal perioperative chemotherapy 460

invasion 148, 553

invasive margin 2010

*in vivo* 324

ionising radiation 889

irinotecan 18, 1591

irreversible electroporation 490

ISET 508

Ki67 1798

kidney cancer 1475

kinase inhibitor 475

KIT 939

*κ*-opioid receptor 1148

KRAS 1406, 1722, 1826

*KRAS* mutation 748

lapatinib 6, 25, 673

laser-induced thermotherapy 1274

LDH 799

LD radiolabelling 1027

let-7 1415

letrozole 1618

leukocyte 585

life change events 1560

Lin28 1415

Lin28B 1415

liposomal doxorubicin 1027

liver metastases 1926

liver resection 53

LMP1 1980

LNCaP 166

localised prostate cancer 1095

locally advanced 61

locally advanced cervical cancer 39

low-risk GTN 1089

lung cancer 447, 575, 867, 1100, 1153, 1346, 1854, 1907

lymphangiogenesis 348

lysate therapy 92

M30 ELISA 1766

M65 ELISA 1766

machine learning algorithm 126

magnesium 1335

magnetic resonance spectroscopy 1638

malignant melanoma 553

malignant pleural mesothelioma 1027

mammography 525

marker protein patterns 1297

mass screening 805, 970

mathematical model 1280, 1742

matrix metallopeptidase 1486

matrix metalloproteinase 1431, 1495

MCM2 1798

mCRC 453

MCV 1166

MDA-7/IL-24 1976

meat 603, 608

meat mutagens 608

MEK 1386

melanocyte 553

melanoma 85, 939, 970, 1134, 1446, 1481

melatonin 1288

menarche 210

menopause 210

mental health 1842

Merkel cell carcinoma 1166, 1314

Merkel cell polyomavirus 1314

mesenchymal stem cells 1901

mesothelioma 575, 1016

meta-analysis 603, 1881

metastasis 133, 148, 174, 348, 1446, 1535, 1668, 1689, 1718

metastatic colorectal cancer 274

metastatic renal cell carcinoma 646, 1587

metformin 1117, 1374

methodology 854

methods 1262

Microarray 1779

microcalcifications 525

micro CT 496

micrometastasis 1675

micronucleus test 780

micronutrients 1891

microRNA 182, 366, 405, 740, 1526, 1826

microRNA expression 768

microsatellite analysis (MSA) 1171

microsatellite instability 1239

microvesicles 896

microvessel 702

Mifamurtide 14

mineralisation 525

miR-143 366

miR-145 366

*miR-1* and *miR-133a* 405

miR-328 1320

mitochondria 1224

mitochondrial DNA 1506

mitotic spindle 307

MMP16 1320

model 1045

molecular clustering 538

molecularly targeted agents 854

molecular marker 562, 909, 1177

MOLM-13 475

monosomy 3 1171

morphology 1875

mortality 1560, 1842

mRCC 284

MRI 1960

Mrna 1790

MRP-1 1224

MTHFR 677 C4T polymorphism 2016

mTOR 876, 1386

mTOR inhibitor 1475

multi-drug resistance 1224, 1395

multi-kinase inhibitor 1722

multiparametric imaging 619

multiple 1068

multiple myeloma 546, 1660

multiple risk-allele GWA replication 389

mumps 1331

mutation screening 1697

MV4-11 475

myxoid liposarcoma 1976

naevus 553

Na,K-ATPase 1807

Nanog 1702

nasopharyngeal carcinoma 206

*NAV3* 517

necrosis 702

needle biopsy 1095

neoadjuvant chemotherapy 53

nephrectomy 279

network analysis 1107

Neuroblastoma 1807

nitrate 608

nomogram 1083

non-Hodgkin’s lymphomas 966, 988

non-seminoma 414

non-small cell lung cancer (NSCLC) 658, 763, 1148, 1953, 1989

normal mucosa 1495

Notch 1953

novel marine-derived compound 1379

Noxa 1660

NPC 1980

NSAIDs 1564, 1772

NSC-134754 1638

NT5E 1446

nurses 651

occupational 1346

OCT-1 1772

Oct4 846

ocular adnexal marginal zone B-cell lymphoma epidemiology 966

oesophageal cancer 210, 702, 711, 955, 1342, 1415

oesophageal squamous cell carcinoma 876, 947

oestrogen receptor 1062, 1205, 1453

OGX-011 1945

olaparib 468

oncogene mutations 939

oncology trials 854

oncolytic HSV 496

one-step nucleic acid amplification assay 1675

oophorectomy 775

orchitis 1331

oropharynx 358

orphan drugs 14

orthotopic model 348, 496

Ouabain 1807

ovarian cancer 70, 189, 333, 482, 596, 629, 633, 1306, 1460, 1779, 1860, 1967

ovarian endometrioma 1205

overall survival 1374

OXi4503 1766

oxidative stress 99, 344

P16 358

P2037 metastasis 1535

p53 397

p57^Kip2^ 482

paclitaxel 633, 1728

PAI-1 366

pancreatic cancer 61, 233, 348, 508, 603, 1004, 1033, 1866, 1934, 1940

paraaortic lymphadenectomy 39

parity 210

PARP inhibitors 18, 1460

partial monosomy 3 1171

pazopanib 629

PC-3 166

pegylated liposomal doxorubicin 633

pelvic lymphadenectomy 39

period analysis 1875

peritumoural 2010

personalised therapy 126

pertuzumab 1779

PFTK1 947

Pharmacokinetics 1598

pharmacokinetics 678

phase 1 468, 619, 629, 673, 1379, 1598, 1766

Phase II 453, 1027

phase III 1268

phase I TAK-285 666

*PHB* 1630 C4T polymorphism 2016

phenformin 1117

phosphoinositol 3 kinase inhibitor 107

physicians 651

PI3 kinase 1386, 1535

PKA 1648

PKC inhibitor 867

plasma 740, 768

plasma C-peptide 1335

platinum agents 1967

PLP2 307

PM00104 1379

Polycomb proteins 243

population-based 1011, 1564

population mixing 1556

population pharmacokinetics 460

positive predictive values 1940

PPAR*γ* 1486

PPAR*γ* agonist 1702

prediction, biological marker 947

predictions 1016

predictive factors 799

preferences 638

primary care 1940

primary healthcare 982

primary invasive breast cancer 383

prodrug therapy 496

progestin 1205

prognosis 133, 189, 297, 719, 904, 909, 923, 931, 1076, 1083, 1171, 1439, 1499, 1551, 1560, 1713, 1798, 1826, 1989

prognostic 1760

prognostic biomarkers 2004

prognostic factors 1095, 1177

prognostic index 1439

prognostic marker 1239

prognostic tool 646

progression-free survival 1587

proliferation 876

prophylaxis 1626

prospective studies 603

prostate cancer 99, 157, 166, 174, 217, 405, 436, 638, 768, 909, 1166, 1689, 1697

protein target identification 254

proteomics 955

proton pump inhibitors 233

*PSCA* 157

psychological stress 1560

PTEN/PI3K/Akt pathway 1367

PTEN 719

purine nucleoside phosphorylase (PNP) 405

qPCR 157, 1605

Q-TWiST 1587

quality-adjusted survival 1587

quality of life (QoL) 638, 646, 826, 1062, 1618, 1027

quiescence 1807

RAD001 876, 1475

RAD51C 1460

RAD51D 1460

radiation 297, 1512, 1953

radiation-induced 297

radiological 633

radiosensitivity 262

radiotherapy 262, 988

random error 1846

randomised, controlled trial 45, 1934

reactive oxygen species 314, 344

receptor tyrosine kinase inhibitor 666, 1598

RECIST 854

recurrence 1160, 1560

recurrence-free survival 2004

regional lymph node metastasis 1735

regional variation 1846

registries 569

Regorafenib 1722

regulatory strategy 14

regulatory T cells 546

relative survival 1846, 1854

release 1989

renal cancer 279, 904

renal cell cancer 1881

renal cell carcinoma 1945

renal pelvis: ureter 1083

renin-angiotensin system 290

response assessment 854

response rates 1021

retinoic acid receptor 77

retrospective review 1374

review 603

RhoGD12 1187

rIL-21 793

risk 1431, 1697

risk modification 996

risk prediction 199

risk profiles 436

RO 5323441 678

ROS 889

RT–PCR 1177, 1314

S-1 1268, 1934

sagopilone 70

salinomycin 99

salivary gland cancer 719

salvage therapy 1591

sample size 1259

saracatinib 1728

sarcoma 297

SATB2 931

second-line chemotherapy 1469

scheduling 858

scoring system 1713

screening 733, 817, 1431, 1753

secondary malignancies 297

self-reporting 1910

seliciclib 482

Selumetinib 858, 1648

seminoma 414

sensitivity analysis 1854

sentinel lymph node 1314, 1675

sentinel node biopsy 39, 1045

serum 768, 1431

serum insulin 227

sexually transmitted disease 1753

side population cells 1320

sigma-2 receptors 693

signal regulated kinase 1453

signet ring cell carcinoma 1668

Sikkim 962

skeletal muscle 1583

skin neoplasms 970

small cell lung cancer 324, 839

small-molecule library screening 254

smoking 447, 1854

snail 1196

SNP 1495

socioeconomic position 988

socioeconomic status 1556

sodium bicarbonate 1280

soft tissue sarcoma 1076

solid tumours 77, 1598

somatic evolution 174

sorafenib 1945

Sox2 1702

sphingosine-1-phosphate 909, 1453

sphingosine 1-phosphate receptor 4 1453

sphingosine kinase 1453

squamous cancer 358

squamous cell carcinoma 107

Src 85, 1728, 1960

Src kinase 1187

stage 1068

statins 685, 1689

stem cells 1702

stemness genes 1702

stratification 1259

stroma 1134, 2004

subjective cognitive function 1618

subsite 1875

sunitinib 646, 1469, 1587

surveys 1021

survival 39, 383, 569, 592, 646, 702, 955, 988, 1083, 1187, 1353, 1439, 1495, 1520, 1564, 1842, 1875, 1907, 2010

survival benefit 638

survivin 763

SVM 1735

symptoms 1940

synergy 333

systematic review 780

systemic inflammatory response 279, 2010

tamoxifen 1618

Taqman 157

targeted agents 619

targeted therapy 148, 1826

taxanes 685, 1967

TB-403 678

T-DM1 6

TDP1 18

temozolomide 858

testicular cancer 414

testicular germ cell tumours 1331

tetraspanin 923

thalidomide 1153

therapy 174, 1117

therapy response 1395

thioredoxin 314

thyroid tumours 562

tissue inhibitors of metalloproteinase 1486

tissue recovery 490

TLS–CHOP 1976

TNF receptor 1866

TNM stage 1713

tolerability 678

tonsillar cancer 1526

topoisomerase I 18

TP53 348

trade-off 638

trastuzumab 6, 25, 32, 1543, 1779

trastuzumab resistance 1367

treatment prediction 931

treatment-related morbidity 297

treatment response 53

triage 817

trichostatin A 116

*TRIP13* 157

triple-negative breast cancer 397, 1107, 1234

triple-negative disease 440

triplet 658

tumour angiogenesis 1214, 1722

tumour biopsy 508

tumour budding 1713

tumour cells 1917

tumour endothelial cells 1214

tumour growth kinetics 854

tumour immunotherapy 92

tumour inflammatory infiltrate 702

tumour invasion front 141

tumour microenvironment 1833

tumour progression 1901

tumour staging 279, 1314

tumour-supernatants 896

tumour suppressor 405

tumour virus 429

TWiST 1587

UAPI 1089

ubiquitin ligase 182

UDP-glucuronosyltransferase 1591

UFT 1268

UKCTOCS 1910

unresectable 61

unresectable colorectal liver metastasis 53

upper-tract urothelial carcinoma 290

uptake 775

urine 768

urothelial carcinoma 1083

uterine serous cancer 1543

utility 638

uveal melanoma 1171

value-based pricing 14

vascular disrupting agent 1766

VCA 206

VEGF 284, 1960

VEGF-targeted therapy 1475

vinorelbine 673

visual counting method 383

VLP 92

vulvar brush 269

vulvar cytology 269

vulvar dysplasia 269

vulvar intraepithelial neoplasia 269

web-based 1076

Weibull model 646

Wittenoom 1016

XCL2 307

xenografts 1117, 1134

xenograft tumours 333

XMRV 1166

young women 1053

